# Difference thresholds in 3D space depend on perceived size, not depth

**DOI:** 10.1007/s00426-025-02181-6

**Published:** 2025-09-30

**Authors:** Aviad Ozana

**Affiliations:** 1 Department of Psychology, Ben-Gurion University of the Negev, Beer-Sheva, Israel; 2 Department of Neurobiology, David Geffen School of Medicine, UCLA, Los Angeles, CA USA

## Abstract

People typically perceive objects to be the same size and shape despite changes in viewing distance, a phenomenon known as object constancy. Recent work suggests that perceptual processing of object shape and size may be enhanced in near space, consistent with similar reports from attentional tasks. These studies, however, have some methodological limitations, as the viewing distance was manipulated using illusory 2D depth cues, for which perceptual processes may differ significantly from those in real three-dimensional (3D) environments. To address this, a series of psychophysical experiments were conducted in which participants’ visual resolution for object length was tested within and outside their peripersonal space. In Experiments 1 and 2, difference thresholds (Just Noticeable Differences, JND) were measured using the Method of Adjustment, while Experiment 3 employed the Method of Constant Stimuli. Experiment 4 tested whether the findings can be extended to 3D stimuli. Results from Experiments 1 and 2 showed that perceived size (PSE) varied with distance. Critically, however, across all experiments, JNDs were modulated by the size of objects and were insensitive to differences in retinal size or viewing distance. These findings suggest that in natural 3D environments, perceptual resolution is constrained by high-level visual representations of object size. More broadly, the study points to potential differences in how perceptual mechanisms operate in 3D versus 2D contexts, consistent with recent findings in the perception and action literature.

**Supplementary Information:**

The online version contains supplementary material available at 10.1007/s00426-025-02181-6.

## Introduction

The images of objects on our retina are constantly shrinking, expanding, and changing shape as we move through the world. The ability to see objects preserving the same size despite these dramatic changes in the images impinging on the retina is called “size constancy” (Boring, 1940; Sperandio & Chouinard, 2015). To compute the size of an object, our brain must integrate the information from the projection of the object on the retina and the object’s apparent distance from the eyes. Without this, our world would seem chaotic and hard to comprehend.

While size constancy provides us with a stable perception of objects’ size across viewing conditions, the extent to which distance affects our ability to detect changes in magnitude is not entirely clear. On the one hand, the relationship between human resolving power and object size is well established in experimental psychology. According to Weber’s law, the smallest detectable change in magnitude (JND or just noticeable difference, measured in physical units) is proportional to the overall magnitude of a stimulus (Fechner, 1860; Stevens, 1946; see Marks & Algom, 1998 for a tutorial review). This principle holds across a range of experimental contexts (for rare departures from Weber’s law, see Carlyon & Moore, 1984; Ganel et al., 2008; see also, Algom, 2021). On the other hand, sensitivity to size differences and similar object-feature judgments has almost always been tested from a fixed distance, leaving open the possibility that the distance from which objects are viewed affects these judgments to some extent.

Two relatively recent studies on this topic have reported an effect of perceived depth on perceptual judgments using 2D illusory depth cues (Ahsan et al., 2022; Blini et al., 2018). For example, in one of these studies, participants were asked to determine, over repeated trials, which of two rectangles embedded in a 2D version of the Ponzo illusion was longer (Ahsan et al., 2022). The researchers found that rectangles that appeared more distant to the observer (placed on the small rectangle in the Ponzo configuration) had larger JNDs compared to when they were perceived as closer (placed on the large rectangle). This may suggest that within these 2D configurations, resolving power is poorer for objects perceived as farther away from the observer, and, more broadly, that perceptual judgments can be sensitive to perceived distance from the targets.

Although the above-mentioned findings align with previous research on spatial attention, which has demonstrated near space superiority for stimuli presented within the Peripersonal Space (PPS) (Fogassi et al., 1996; Tseng & Bridgeman, 2011; Di Pellegrino & Ladavas, 2015), some ambiguity surrounds the use of 2D depth cues in recent work (Ahsan et al., 2022; Blini et al., 2018; also see, Ahsan et al., 2025; Britt et al., 2025; Dureux et al., 2021; Haponenko et al., 2024; Zafarana et al., 2024, for related work on spatial attention). One difficulty lies in the use of size-contrast illusion to manipulate apparent depth. These configurations not only affect the object’s perceived distance but also its perceived size (Gillam, 1980; Gregory,^1968^). Indeed, an alternative interpretation is that target objects may not have maintained size consistency across different viewing conditions: objects perceived as more distant appeared larger, resulting in increased JNDs, in line with Weber’s Law (Marks & Algom, 1998). Another limitation involves the use of 2D instead of 3D depth cues. Recent findings suggest that perceptual judgments, as well as visuomotor processing, differ significantly for 2D and 3D environments (Ozana & Ganel, 2018; Ganel et al., 2020; Ozana, Berman, & Ganel, 2020; Plumert et al.,^2005^; Snow & Culham, 2021; Yildiz et al., 2024). A possible reason for these differences is the absence of rich monocular and binocular depth cues in 2D configurations, which may disrupt size estimations. Consequently, it remains unclear whether these findings could be generalized to conditions involving real objects and actual changes in viewing distance.

To address this open question and the concerns mentioned above, a series of psychophysical experiments were conducted in which stimulus size and (3D) viewing distance were manipulated independently. To maximize a potential effect of distance while ensuring that objects remained easily identifiable and binocular cues were preserved, two distinct viewing positions were selected: one within near space (35 cm) and one beyond the typical boundary of PPS (65 cm), for which previous work has shown decreased performance (Holmes, 2012; Kandula et al., 2017; Serino, 2019). This design also enabled us to dissociate retinal and perceived size by measuring the just-noticeable difference (JND) between objects of different physical sizes that projected the same visual angle onto the retina when presented from both distances (e.g., 3.5 cm at 35 cm and 6.5 cm at 65 cm). Thus, we could determine whether size-related differences in JND are influenced by the perceived size, its distance from the observer, or changes in the size of the image projected onto the retina.

In addition to the difference threshold or JND, we also measured the PSE (Point of Subjective Equality) for each object presented from a close or distant view, which was taken to reflect the perceived size of the object. In Experiments 1 and 2, we compared judgments of object lengths using the psychophysical procedure known as the Method of Adjustment (e.g., Baird & Noma, 1978). In Experiment 3, we employed a different psychophysical method, the Method of Constant Stimuli, with the same set of stimuli, again presented at varying viewing distances. Finally, in Experiment 4, we examined whether the findings extend to 3D stimuli.

## Experiment 1

### Methods

#### Participants

Twenty-six participants (average age 23.2, SD = 0.98, 3 males) participated in the experiment for course credit. All the participants provided informed consent prior to their participation, approved by the institution’s ethics committee Review Board. A priori power analysis conducted using G*Power analysis software (Faul et al., 2007) for a 2 × 2 repeated-measures ANOVA (*α* = 0.05, 1–*β* = 0.80) indicated sufficient statistical power to detect a moderate effect size of *f* = 0.30. This effect size was smaller than what has been reported in previous studies on this topic (Ahsan et al., 2022; Blini et al., 2018). A correction for outliers was conducted for each participant’s data, based on the mean score for the judgments of object length. A judgment on a trial that was three SDs larger than the mean was removed from further analysis. This correction resulted in the removal of less than 2% of the trials in total.

#### Design

As already mentioned, JNDs in this experiment were measured using the Method of Adjustment (Baird & Noma, 1978; Mathôt et al., 2012). In this method, the observer adjusts the size of a stimulus under their control to match the size of a standard stimulus presented. While under basic conditions, most psychophysical procedures provide comparable results (Baird & Noma, 1978; Perssey, 1977), a significant advantage of this procedure is that it enables participants to reflect their perception of the standard stimulus’s size directly. This makes it especially useful for examining within-subject effects in multivariate experimental design (Chen et al., 2019a, b; Rolland et al., 2002), and, importantly for the current study, for gauging PSE and JNDs independently of the standard’s viewing position. Hence, this allowed us to investigate distance-related effects for differently sized objects while dissociating retinal image size from perceived size, which would be more challenging to achieve using the Method of Constant Stimuli (see Experiment 3).

In a typical experiment, the standard and adjustable stimuli are presented at the same viewing distance throughout the experiment. Here, the position of the standard stimulus was changed between blocks, while the position of the adjustable stimulus remained constant at a different distance from it. The participant was seated in front of two 15-inch LCD monitors (ASUS VivoBook, resolution 1920 × 1080 pixels, refresh rate 50 Hz), with their head resting on a chinrest, allowing them to maintain a fixed viewing distance (and retinal size) throughout the experiment. The left monitor displayed the standard stimulus (i.e., fixed in size) and was either 35–65 cm from the participant’s eyes.

**Fig. 1:**
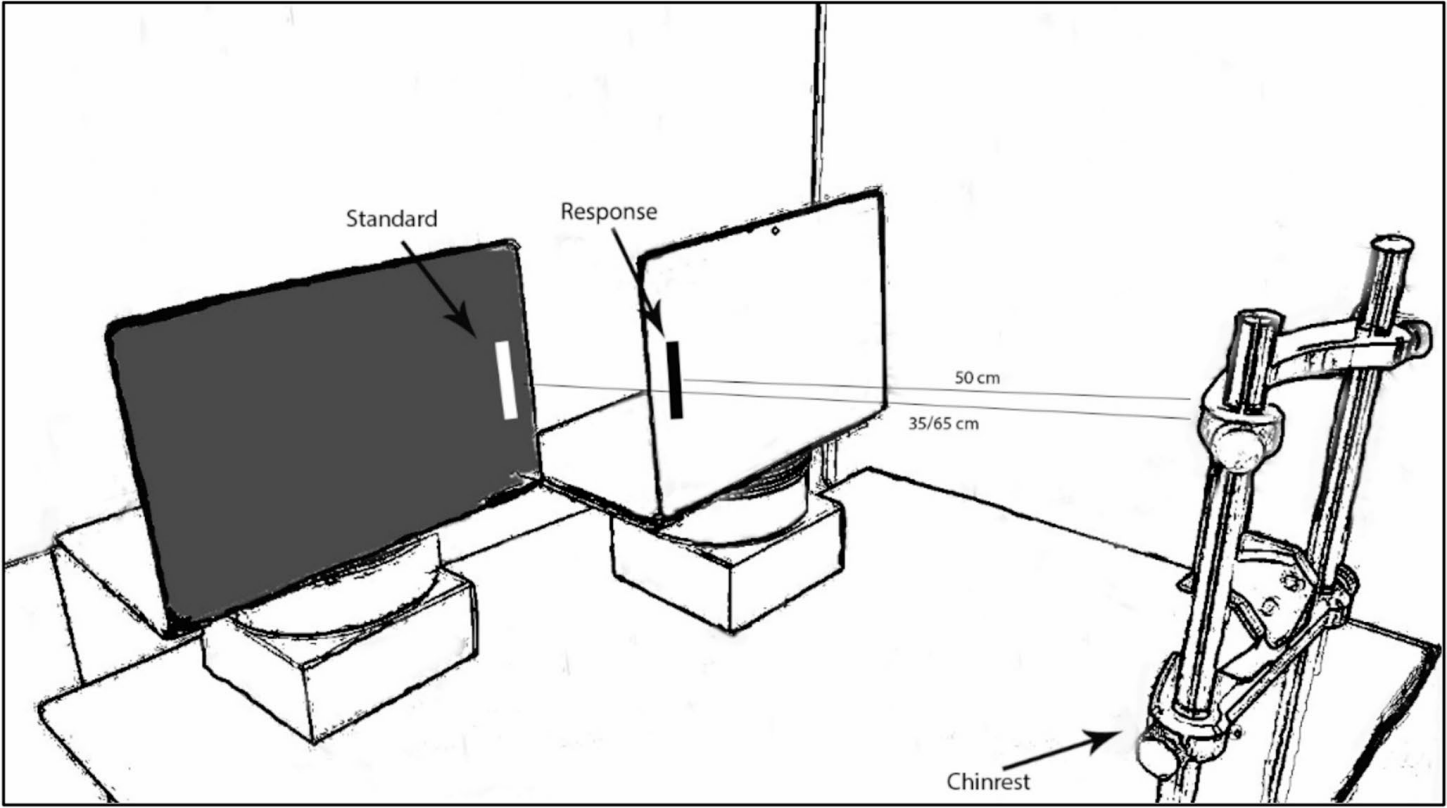
Illustration of the setting of Experiment 1. The participants reproduced the length of a standard stimulus of either 3.5–6.5 cm, presented at a distance of either 35–65 cm, by setting the length of an adjustable response line under their control

These distances were ideal since they corresponded to locations within or beyond PPS, respectively (Serino, 2019). Furthermore, this allowed us to present objects that produce the same retinal size while remaining relatively large and identifiable. The location of the right monitor, displaying a stimulus to be adjusted to match the size of the standard, was held constant at 50 cm. The horizontal distance between the centers of the two stimuli was 5 cm. The adjustable stimulus was a black rectangular object which was initially 1 cm shorter or 1 cm longer than the standard. Standard targets were vertical white rectangular bars 3.5–6.5 cm long (1 cm in width), presented against a black background (see Fig. 1). Note that when the 3.5 cm standard was presented at the near location (35 cm), it had the same visual angle on the retina as a 6.5 cm standard presented from 65 cm distance (5.72° in both cases). See Table 1 for the visual angles subtended by the short and long standard stimuli at the two distances.

To keep participants engaged in the task, we also presented two additional foil standards of 4.5 cm, and 5.5 cm in length. OpenSesame (Version 3.2) and MATLAB (Version 9.0, The Mathworks, Natick, MA) were used to control the trial sequence and stimulus presentation.

**Table 1:** Visual angle of the standard stimuli in Experiment 1. Note that the smaller object in the near distance produced the same visual angle as the bigger object presented at the far distance. The position of the adjustable stimulus was fixed at a distance of 50 cm from the observer.

Viewing distance/Physical size	3.5 cm	6.5 cm
35 cm (PPS)	**5.72°**	10.6°
65 cm (EPS)	3.43°	**5.72°**

#### Procedure

The participants changed the size of the response stimulus under their control by using the computer mouse wheel and ended by pressing the left key of the mouse. Each click of the wheel changed the length of the response stimulus size by 1 mm. There was no time limit for performing the judgment. After each trial, a fixation point was presented for 500 ms on the screen of the standard stimulus.

Each participant performed the task in two blocks of trials, one in which the standard stimulus was presented at the near (35 cm) distance and one in which the standard stimulus was presented at the far (65 cm) distance. Each of these blocks contained 24 experimental trials (10 trials for the 3.5-cm standard 10 trials for the 6.5-cm, and 2 trials each for the foil stimuli), for a total of 48 trials. The order of near and far blocks was counterbalanced across participants, and the trial order within blocks was pseudo-randomized.

#### Data analysis

The JND for the length of each standard was defined by the standard deviation of the responses (Baird & Noma, 1978; Marks & Algom, 1998). The PSE was the mean response. JND and PSE for each participant were calculated separately for the 3.5-cm standard and the 6.5-cm standard at each of the two viewing distances (35 cm and 65 cm). The effect of distance and size was analyzed by repeated-measures ANOVA. Post hoc comparisons of JND and PSE values were to be conducted if significant effects were observed in the ANOVA.

Additional specific comparisons were used to test directly whether retinal size affects visual resolution; that is, we computed the mean difference in JND by subtracting each participant’s JND for the near 3.5 cm object from the mean JND for the far 6.5 cm object (equal retinal image size). We then compared it to the mean difference in JND between the near 6.5 cm object and the far 3.5 cm object (different retinal image size). The absence of a difference in JND would indicate that retinal-image size does not, by itself, affect the perceptual resolution of length.

To further examine the possible absence of an interaction between distance and object size, a Bayesian repeated-measures ANOVA was performed using JASP software version 0.19.3 (JASP team, 2025) with default Cauchy priors of *r* =.707, along with separate Bayesian paired-samples t-tests for each object size. Note that the main effect of distance was not further analyzed, given that the expected Bayes Factor in favor of the null hypothesis (BF₀₁) was in the range of hundreds, providing between very strong to extreme evidence against the main effect.

Lastly, to further examine a potential relationship between perceived size and perceptual resolution, we conducted a multiple linear regression analysis. Each participant’s mean PSE, viewing distance, and their interaction were included as predictors. JND was the dependent variable.

#### Results

**Fig. 2:**
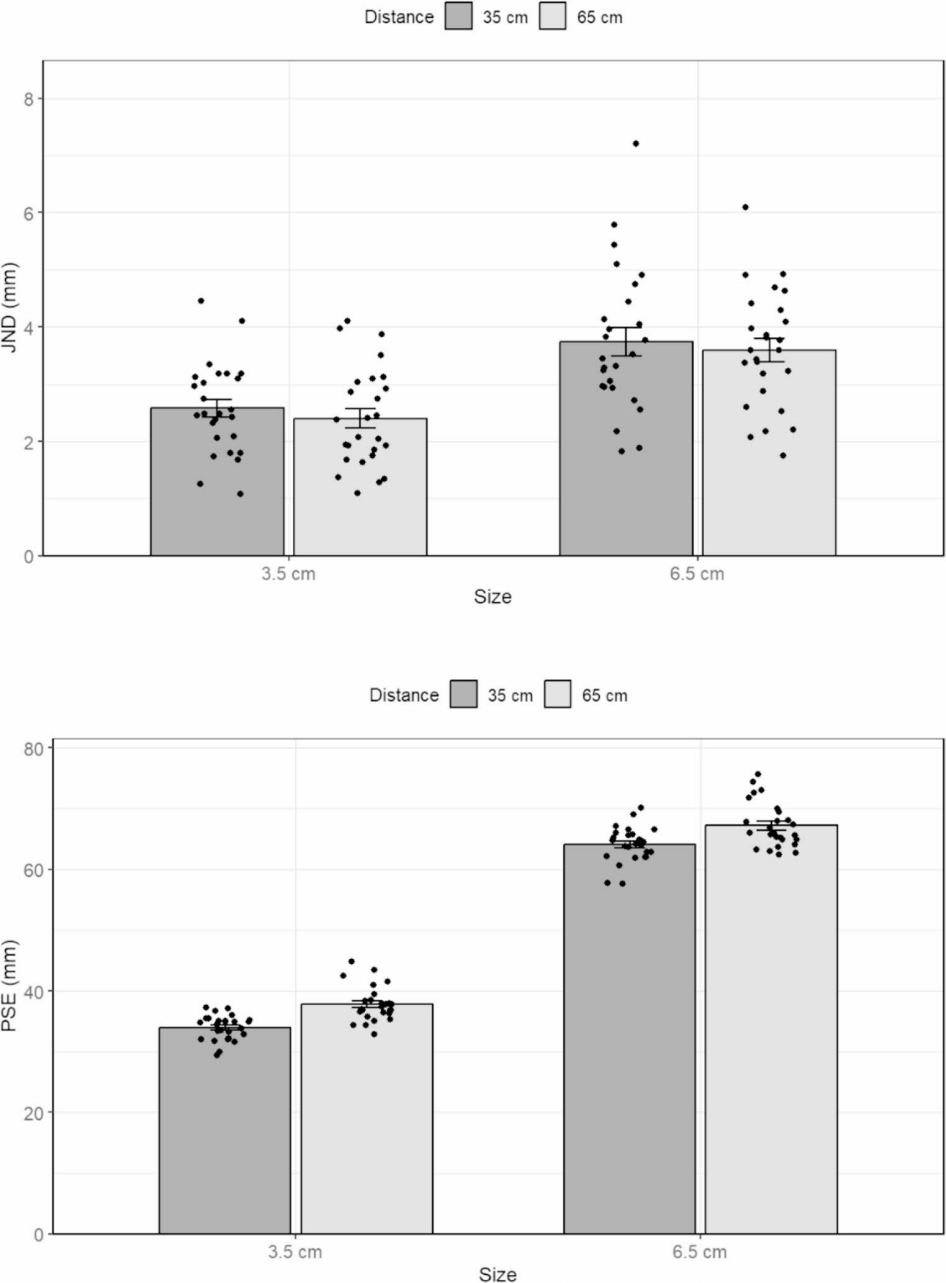
The results of Experiment 1. Top panel: The just noticeable difference (JND) for two standards (3.5 and 6.5 cm) presented at two different distances from the observer (35 and 65 cm). Bottom panel: The Point of Subjective Equality (PSE), for two standards at two distances. Error bars represent the standard error of the mean

Figure 2 depicts the values of the JND and PSE for the two standard stimuli presented at different viewing distances. The results suggest that JND was not affected by the distance from the observer or by the visual angle projected on the retina.

To test the effect of distance on the JND, we used a 2 × 2 repeated measures ANOVA with physical size (3.5 vs. 6.5 cm) and distance from the observer (35 vs. 65 cm) as factors. The main effect of distance was not significant, *F(1*,* 25)* = 0.8, *p* =.36. There was, however, a significant main effect of size, *F(1*,* 25*) = 57.9, *p* <.*001*,* η*_*p*_^*2*^ = 0.69, which indicates that, in line with Weber’s Law, the JND was larger for the longer object than it was for the shorter one (means of 3.6 and 2.4 mm, respectively). The interaction between distance and size was not significant, *F*(1, 25) = 0.15, *p* =.90. Further, the Bayesian repeated-measures ANOVA, aimed at testing the probability of the null hypothesis, revealed that the model that included the interaction between distance and size, BF_01_ = 10.22, was approximately 10 times less likely than the model including only size. Note that separated Bayesian paired-samples t-tests for each object size also supported the null hypothesis (BF₀₁ = 2.65, BF₀₁ = 4.25, for the small and large objects, respectively), providing an anecdotal to moderate evidence, even when the interaction was disregarded.

Furthermore, a comparison between standards of the same retinal image (small-near and long-far 6.5) and standards with different retinal images (large-near and small-far) did not result in a difference in JND, *t(25)* = 0.9, *p* =.36. This indicates that retinal size alone did not affect the difference threshold.

Surprisingly, unlike its null effect on the JND, distance was found to have a significant effect on the PSE, *F*(1,25) *=* 18.8, *p* <.001,* η*_*p*_^*2*^ *=* 0.43. As can be seen in Fig. 2b, objects presented at the near viewing distance were perceived to be shorter than at the far distance (49 mm and 52.5 mm, respectively, averaged across both object sizes). In hindsight, this result is consistent with previous literature showing perceived size varies with viewing distance, possibly as a consequence of accommodation and convergence at close distances (Leibowitz & Moore, 1966; Ono et al., 1974 ). As one would expect, despite this bias, participants were sensitive to the actual length of the object, [*F(1*,*25) =* 1,349.9, *p* <.001,* η*_*p*_^*2*^ *=* 0.99 (35.8 and 65.8 mm, respectively). The interaction between distance and size did not reach statistical significance, *F(1*,* 25) =* 2.4, *p* =.12.

Lastly, note that the multiple linear regression model using each participant’s PSE and viewing distance as the predictors of JND produced comparable results to the main findings here. The model, *F*(3, 100) = 10.88, *p* <.001, *R²* = 0.246, revealed a significant effect of PSE, *b* = 0.039, *t*(100) = 4.22, *p* <.001, indicating that larger perceived sizes were associated with higher JNDs. In contrast, viewing distance did not significantly predict JND, *b* = − 0.105, *t* = − 0.15, *p* =.880. The interaction between PSE and distance *b* = –0.004, *t* = −0.28, *p* =.783, was also not significant.

The results from Experiment 1 suggest that the JND is based on the size of the object, irrespective of distance or the size of the visual angle it subtends on the retina. The goal of the next series of experiments was to further evaluate and extend this conclusion. One possible limitation of the design used in Experiment 1 is that the adjustable stimulus was always presented at a fixed distance from the participant. Therefore, in Experiment 2, we manipulated the viewing distance for both the adjustable and the standard stimulus. Specifically, when the standard stimulus was presented at the near viewing distance, the response stimulus was presented at the far distance, and vice versa.

## Experiment 2

### Methods

#### Participants

Sixteen participants (average age = 22.2, SD = 1; 3 males) participated in Experiment 2 for course credit, consistent with a priori power analysis recommendations for detecting f = 0.30 in a 2 × 2 repeated-measures ANOVA (Faul et al., 2007). The smaller sample size was further supported by the large effect size observed for object size in Experiment 1, indicating that sufficient statistical power could be achieved with fewer participants.

#### Design and procedure

The design was like that of Experiment 1 (see Fig. 1), except that now we also manipulated the position of the right-hand screen, displaying the response stimulus. In two separate blocks of trials, the to-be-adjusted stimulus was presented at a viewing distance of either 35–65 cm from the participant. The position of the response stimulus was always opposite to that of the standard (i.e., 35 cm when the reference position was 65 cm, and vice versa). Again, each participant was tested in two separate “near” and “far” blocks (block order was counterbalanced), each containing 24 experimental trials (including foil stimuli) or 48 trials in total. The results from one participant, who did not follow the experimental procedure, were removed from the analysis. The correction for outliers resulted in the removal of less than 2% of the trials.

#### Results

**Fig. 3:**
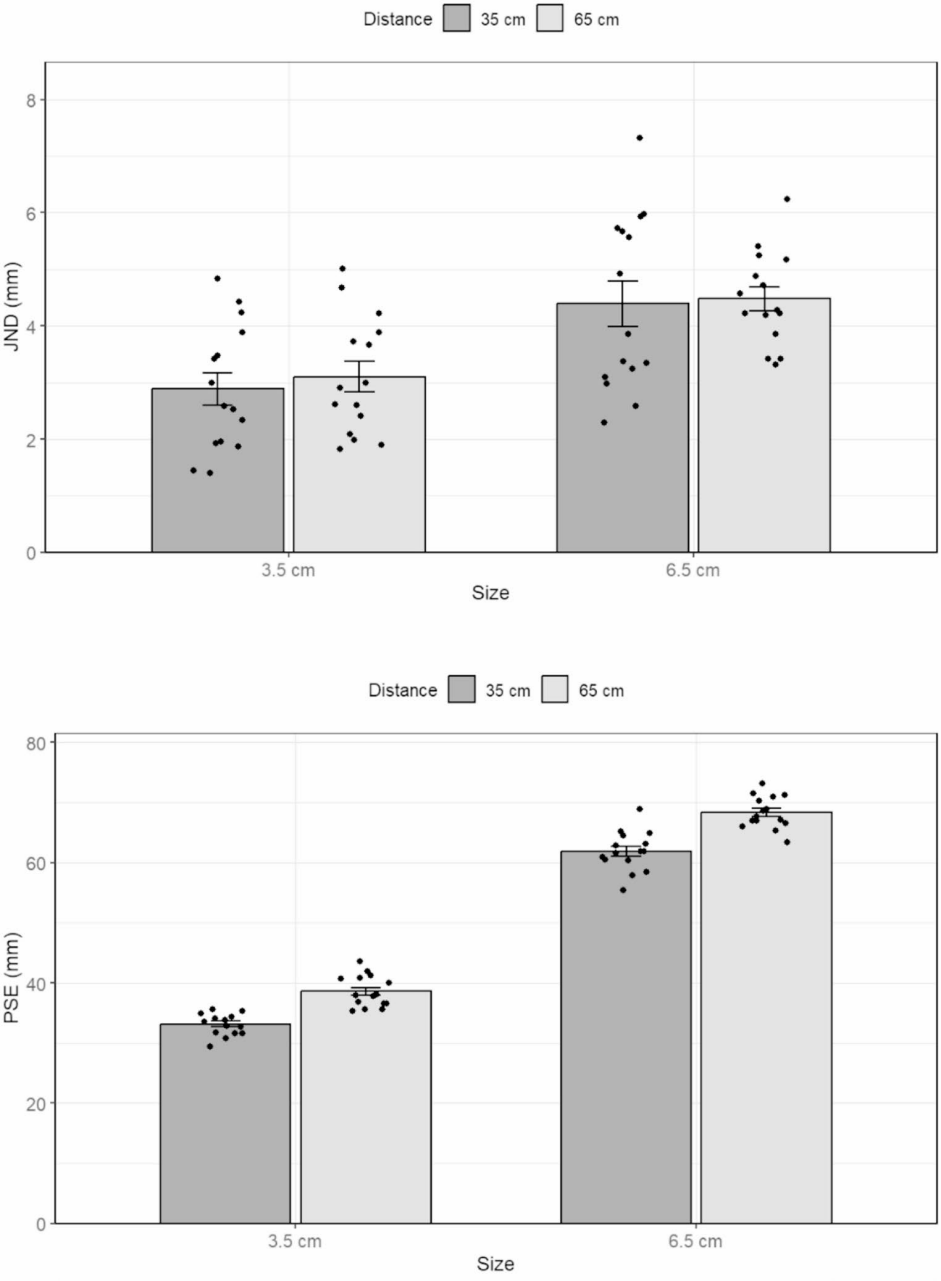
The results of Experiment 2 for both the JND (top panel) and the PSE (bottom panel). In Experiment 2, the distance of the screen presenting the adjustable stimulus also varied. Error bars represent the standard error of the mean

As in Experiment 1, the JND was unaffected by viewing distance (Fig. 3). A repeated-measures ANOVA with respect to the JND, using distance and size as independent variables, did not reveal a main effect of distance, F(1,14) = 0.2, *p* =.65. By contrast, the significant main effect of size, *F(1*,*14) =* 36.8, *p* <.001,* ηp2 =* 0.72, indicated that JNDs were sensitive to the actual length of the object (2.9 mm, 4.4 mm for the short and long standard, respectively). There was no interaction between distance and size, *F(1*,*14) =* 0.07, *p =*.79. As in Experiment 1, an additional 2 × 2 Bayesian repeated-measures ANOVA provided support for the null hypothesis of no interaction between distance and size. A model that includes the interaction between distance and size was approximately 6.9 times less likely (BF₀₁ = 6.939) than the best-fitting model, which only included size. As before, a separate Bayesian paired-samples t-test of each size provided moderate evidence for the null (BF₀₁ = 3.24, BF₀₁ = 3.75, for the small and large objects, respectively).

A specific planned comparison between the standards with and without the same retinal size was not significant, *t(14)* = 0.4, *p* =.65. This result supports the notion that discrimination of size was based solely on the perceived size of the objects.

Also in line with the Experiment 1 results, an analysis of the PSE data using a repeated-measures ANOVA showed a main effect of distance, *F(1*,*14) = 58.5*,* p <.*001, *ηp2 =* 0.80, indicating that the PSE was smaller for closer objects (47.4 mm and 53.4 for near and far standard objects, respectively, averaged across the two object lengths). There was a significant main effect of size, *F(1*,*14) =* 6424, *p* <.001, ηp² = 0.99 (35.8 mm and 65.1 mm, respectively). There was no interaction between distance and size, *F(1*,* 14) = 1.7*,* p =*.20 (Fig. 3b).

Finally, as before, in line with the primary analysis of actual sizes, a multiple linear regression model, *F*(3, 56) = 8.46, *p* <.001, *R²* = 0.312, adjusted *R²* = 0.275, revealed a significant effect of PSE, *b* = 0.059, *t*(56) = 4.11, *p* <.001, indicating that larger perceived size was associated with increased JNDs. Viewing distance was not a significant predictor, *b* = 0.825, *t*(56) = 0.79, *p* =.436, in this model, nor was the interaction between PSE and distance *b* = − 0.019, *t*(56) = − 0.96, *p* =.342.

The results of Experiment 2 replicate and extend those of Experiment 1. More specifically, the results suggest that the earlier observations could not be accounted for by the fixed position of the reference stimulus. As before, our findings indicate that JND was only modulated by size. Interestingly, viewing distance again had a significant effect on PSE, which aligns with previous literature.

In Experiment 1–2, viewing distance did not affect the size of the JND. The goal of Experiment 3 was to further validate and extend these findings by using a different psychophysical scaling method: the Method of Constant Stimuli. One potential advantage of this method is that it is based on simple, forced-choice size judgment that can potentially minimize response errors and increase statistical power (Baird & Noma, 1978).

## Experiment 3

### Method

#### Participants

Twenty participants (average age = 23.2, SD = 1, 5 males) participated in Experiment 3 for course credit and were randomly assigned to either the short or long standard condition (10 participants in each group). Sample size represents a priori power-analysis recommendation for detecting f = 0.35 in a 2 × 2 mixed model ANOVA (Faul et al., 2007). This effect size is smaller than those observed in Experiments 1 and 2. Also note that the statistical power is typically greater in the Method of Constant Stimuli (Baird & Noma, 1978).

#### Design and procedure

JNDs were measured using the Method of Constant Stimuli. In this method, two stimuli are presented in each trial (the standard and the comparison), and the participant selects the larger stimulus. In the current study, the standard stimulus was fixed to either 3.5–6.5 cm in length. Twelve different comparison stimuli were used for each standard, six were smaller and six were larger in length. The incremental change in length was 1.75%, proportional to each standard size (0.6 mm and 1.1 mm in constant intervals for the 3.5 and 6.5 cm standard, respectively). The participant indicated the larger object using the left or right arrow keys on the keyboard.

**Fig. 4:**
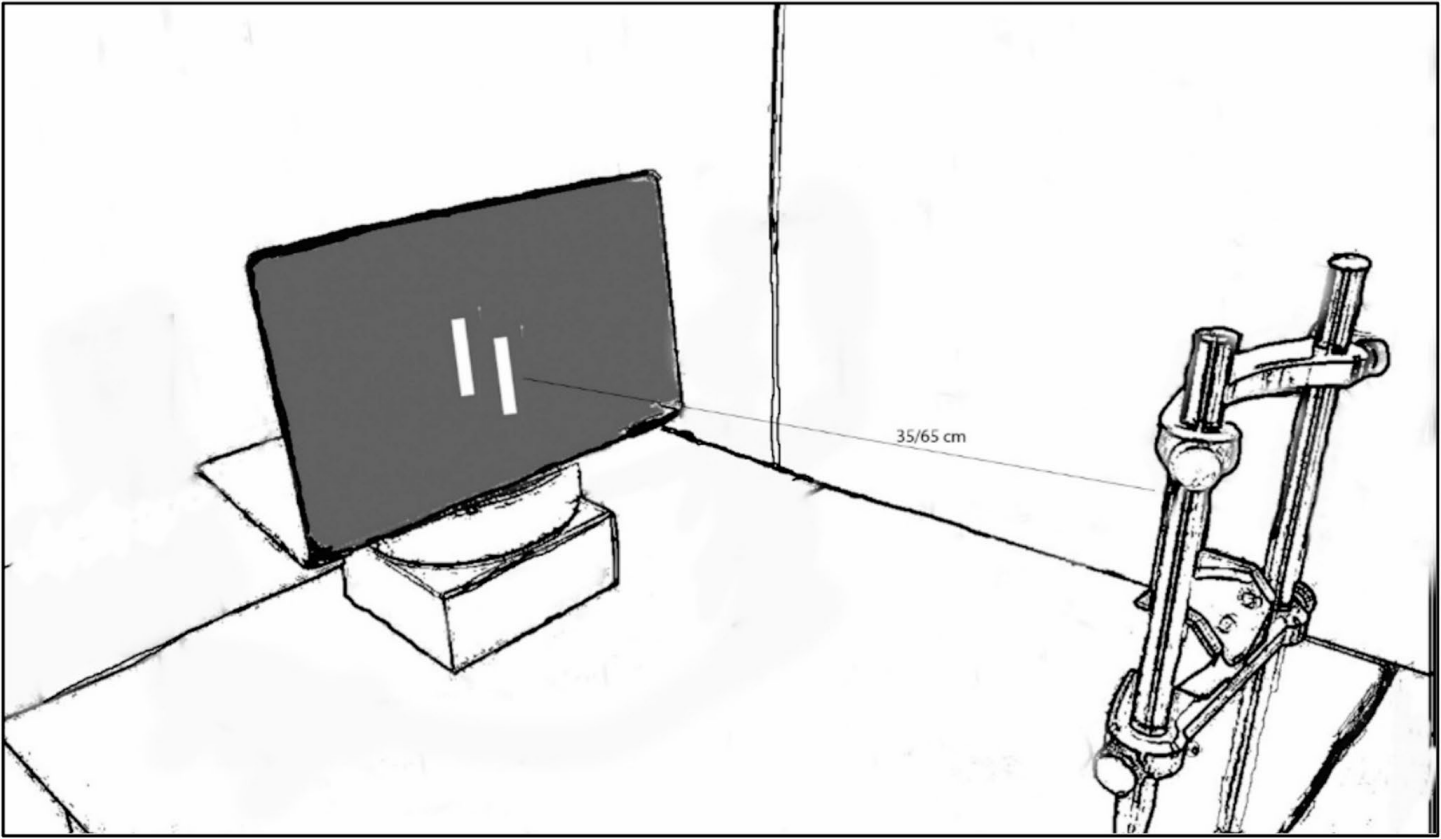
Illustration of the setup of Experiment 3. The participants selected the longer of the two stimuli presented on the screen by pressing the left or the right arrow key on the keyboard. In each trial, one of the lines was the standard, and the other was one of 12 comparison lines

The stimuli were presented on a single 15” monitor. The monitor was presented at a viewing distance of either 35–65 cm. The standard stimulus was positioned in one of four locations around the center of the screen (upper-right, upper-left, lower-right, lower-left), the comparison stimulus always appeared counterclockwise to it (see Fig. 4; for a similar design, see, Zitron-Emanuel & Ganel, 2018).

A fixation point was presented for 500 ms between each trial. Each combination of target and comparison stimuli was repeated 10 times. Trial order was pseudo-randomized. A mixed block design was used, and each group underwent two experimental blocks of the same size from different distances (120 trials X 2 viewing distances, for a total of 240 trials). Block order was counterbalanced.

#### Data analysis

To derive each participant’s JND under each of their experimental conditions, we first calculated the mean proportion of “longer” responses for each comparison stimulus. The resulting data was then fitted to a sigmoid function of the form 1/(1 + exp(-(x-A)/B) using Matlab’s *fit* function. The goodness-of-fit (GOF), Constant Error (CE), and JND were then computed based on the fitted values (see Table 2). The PSE was defined here by the value corresponding to 50% correct discrimination. JND was calculated by subtracting the value of 75% “longer” responses from the value of the 25% “longer” responses and dividing the results by two (for a similar analysis, see Zitron-Emanuel & Ganel, 2018).

We set an exclusion criterion of participant data with a GOF below 0.7. GOF values, however, were high across all participants’ conditions (mean = 0.95, SD = 0.04), and all participant data were retained for further analysis.

Note that since the target and comparison stimuli were presented at the same distance from the participant, the design did not allow for testing potential effects of distance on PSE independently of retinal size.

#### Results

**Fig. 5:**
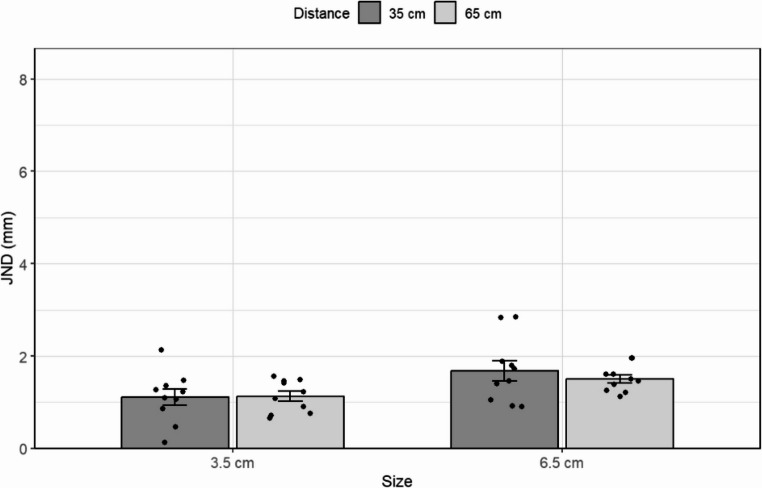
The results of Experiment 3: The JND as a function of the standard stimulus size and distance from the observer. Error bars represent the standard error of the mean

The individual JND, CE, and GOF values, are presented in Table 2. JND was again associated with the physical size of the standard stimulus and was unrelated to viewing distance (see also Fig. 5).

A mixed-model ANOVA with size as a between-subject factor and distance as a within-subject factor was conducted on the JND data. The main effect of viewing distance, *F(1*,*18) =* 0.39, *p =.*55, was not significant. There was a significant main effect of size, *F(1*,*18) =* 6.3, *p =.*02, *ηp2 =* 0.26, with a larger JND for the longer objects (1.1 mm and 1.5 mm for the short and long standard, respectively). However, the interaction between distance and size was not significant, *F(1*,*9) =* 0.75, *p =*.39. A mixed-model Bayesian ANOVA provided moderate evidence for the null hypothesis, with a model including the interaction term being approximately 5.4 times less likely than the best-fitting model, which only included object size (BF₀₁ = 5.414). Disregarding a potential interaction between distance and size yielded a similar pattern. Separate Bayesian paired-samples t-tests within each object size provided anecdotal to moderate evidence for the null (BF₀₁ = 3.18, BF₀₁ = 2.33 for the small objects and the large objects, respectively).

The specific comparison between the difference for the standards with the same retinal image size and that for the standards with different retinal sizes was again non-significant *t(18)* = 0.6, *p* =.55, indicating that differences in retinal size by themselves did not affect the JND.

**Table 2:** Psychometric function parameters and model fit for individual participants under 35 cm and 65 cm viewing conditions, in the 3.5 cm (**a**) and 6.5 cm (**b**) standard stimulus groups. In both groups, participants' JNDs varied as a function of stimulus size but not viewing distance

	CE		JND		GOF	
ID	35 cm	65 cm	35 cm	65 cm	35 cm	65 cm
a.	3.5 cm					
1	0.02	0.44	1.36	0.76	0.90	0.97
2	0.13	0.09	2.13	1.42	0.91	0.88
3	0.36	−0.46	1.47	1.46	0.94	0.95
4	0.04	−0.85	0.86	0.91	0.98	0.97
5	−1.05	−0.36	0.46	0.65	0.87	0.99
6	0.90	0.46	0.08	0.71	1.00	0.99
7	−0.46	0.18	1.09	1.08	0.91	0.99
8	0.80	0.80	1.23	1.23	0.93	0.89
9	−0.97	−0.80	1.06	1.49	0.90	0.88
10	0.02	−0.01	1.27	1.56	0.91	0.90
mean	**−0.02**	**−0.05**	**1.10**	**1.12**	**0.93**	**0.94**
b.	6.5 cm					
1	−0.69	−0.07	1.8	1.61	0.94	0.99
2	−0.39	−0.14	1.72	1.46	0.94	0.97
3	−0.29	−0.27	2.83	1.38	0.79	0.98
4	−0.24	0.18	0.92	1.21	0.95	0.98
5	0.22	0.13	0.90	1.112	0.99	0.98
6	0.84	0.11	1.40	1.25	0.99	0.99
7	0.22	0.05	1.89	1.50	0.96	0.95
8	−0.21	−0.41	1.05	1.60	0.98	0.95
9	0.60	0.02	2.85	1.96	0.92	0.93
10	−0.59	0.27	1.46	1.96	0.91	0.94
mean	**−0.05**	**−0.01**	**1.68**	**1.50**	**0.94**	**0.97**

Unlike the previous experiments, a mixed-model ANOVA on the PSE data revealed no significant main effect of distance, *F*(1,17) = 0.153, *p* =.701. There was a significant main effect of object size, *F*(1,17) = 21981.5, *p* <.001, η² = 0.99. The interaction between viewing distance and size was not significant, *F*(1,17) = 0.020, *p* =.888.

Finally, the multiple linear regression analysis, *F*(3, 36) = 3.20, *p* =.035, *R²* = 0.210, adjusted *R²* = 0.145, revealed that PSE values significantly predicted JNDs, *β* = 0.538, *t*(36) = 2.57, *p* =.015, with higher perceived size associated with larger JNDs. Neither distance, *b* = 0.244, *t* = 0.48, *p* =.637, nor the interaction between PSE and distance, *b* = − 0.068, *t* = − 0.34, *p* =.528, were significant predictors.

The results of the JND analysis in Experiment 3 replicate and extend those of Experiments 1 and 2 using a different psychophysical scaling method. Note, however, unlike before, viewing distance did not affect the PSE in this experiment. A potential explanation is the scaling method employed in Experiment 3, which utilized force-choice, potentially reducing response errors (Baird & Noma, 1978).

In Experiments 1–3, the stimuli were presented on a 2D plane of a horizontal LCD monitor. The goal of Experiment 4 was to test whether the results also extend to real 3D objects presented on the tabletop in front of the participant. Note that such viewing condition also provides for a richer perspective about the shape and other dimensions of the object. As mentioned in the introduction, previous studies reported significant differences in the visual processing of 2D images and 3D objects (Snow et al., 2011; see also vision-for-action, Ozana & Ganel, 2018, 2019, 2020a).

Another modification in Experiment 4 was the use of three rather than two different-size objects, allowing us to test effects of viewing condition on a wider range of objects.

## Experiment 4

### Method

#### Participants

Eighteen participants (average age = 23.3, SD = 0.9, 4 males) participated in the experiment for course credit, consistent with a priori power-analysis recommendations for detecting an effect size of f = 0.25 in a 3 × 2 repeated‐measures ANOVA (Faul et al., 2007).

#### Design and Stimuli

**Fig. 6:**
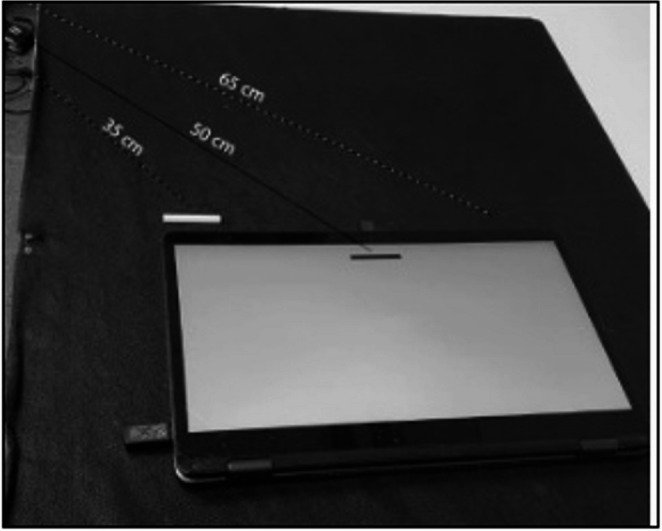
Side view of the experimental setup in Experiment 4. The participants adjusted the length of the comparison 2D rectangle presented on the screen to match the length of a standard real 3D object

Three 3D rectangularly-shaped plastic rods of different lengths (3.5, 5, or 6.5 cm in length, 1 cm wide, 1 cm in height) were used as standards. Since three different exemplars were presented, we did not present additional foil stimuli in the current design. The standard stimulus was placed on the tabletop, 35 cm or 65 cm from the participant’s eyes (average point of view was determined based on pilot data). Similar to the design of Experiments 1 and 2, the adjustable stimulus was a 2D black rectangle presented on a vertical 15” LCD display placed to the right of the 3D object (Fig. 6). It was 1 cm longer or shorter than the standard stimuli. The participant’s viewing distance from the response stimulus was 50 cm (fixed position). The distance between the center of the standard 3D stimulus and the center of the 2D response stimulus was 15 cm (measured at an angle of 60**°** for the close position and 300**°** for the far viewing condition**)**. To allow comfortable viewing, no chinrest was used in this experiment, and participants could move their heads freely.

Each of the standards was presented 10 times at each viewing distance (close/far), and in one experimental block (3.5 cm, 5 cm, 6 cm X 10 repetitions X 2 viewing distances, 60 trials in total). Viewing distance and size were pseudo-randomized within the block. The results of one participant, who did not follow the experimental procedure, were excluded from further analysis. Less than 2% of the trials were considered outliers and were removed from the analysis. Otherwise, the design and procedure of the experiment were like those used in Experiment 1.

#### Results

**Fig. 7:**
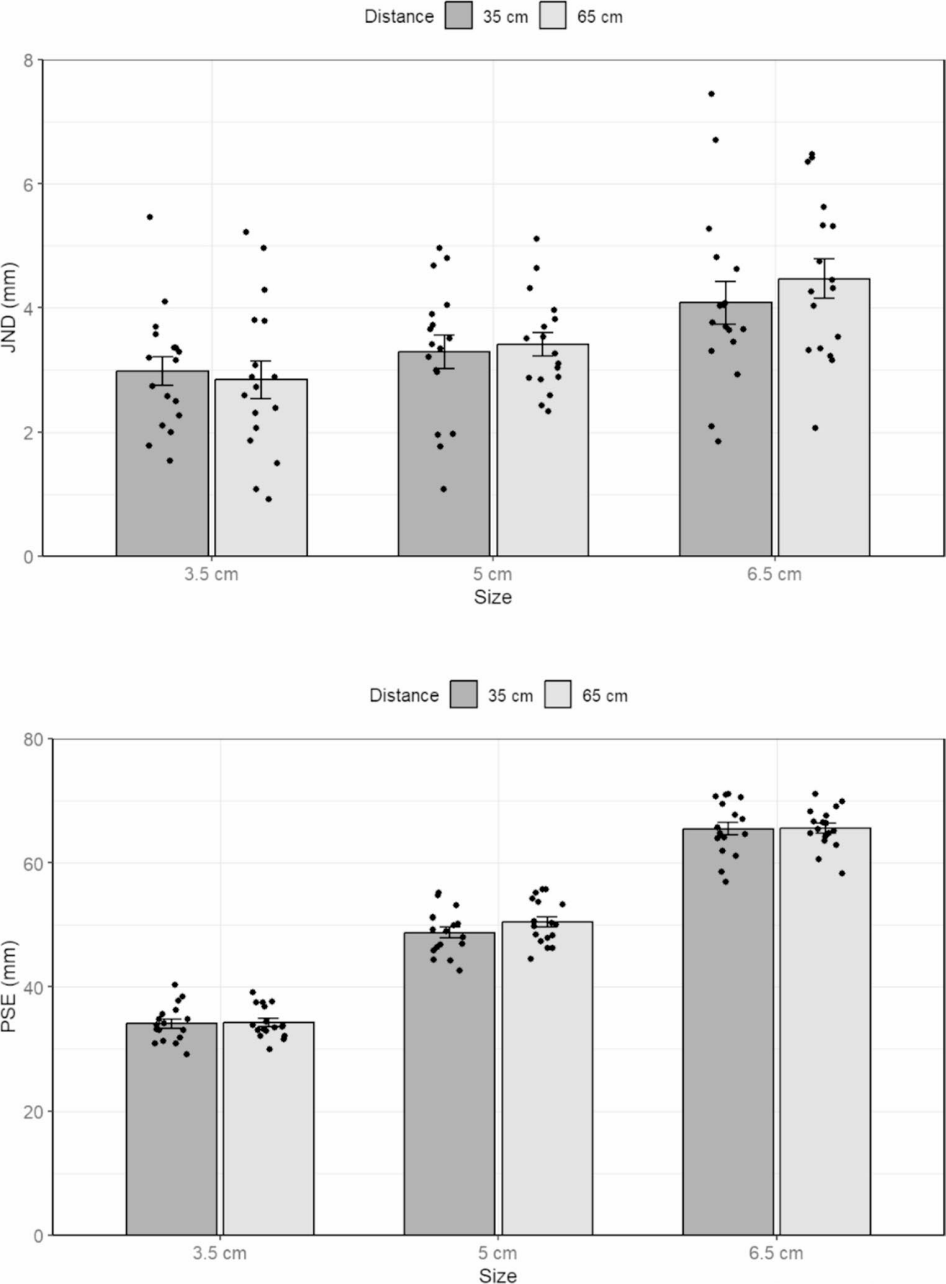
The results of Experiment 4. Top panel: The JND as a function of the size of a real object and of viewing distance. Bottom panel: The PSE as a function of the same variables. Error bars represent confidence intervals in repeated measures ANOVAs (Jarmasz & Hollands, 2009)

The values of the JND and PSE for the different standards viewed at different distances are presented in Fig. 7. Again, the results show that the JND was based on the physical size of the objects rather than distance or visual projections on the retina. A repeated-measures ANOVA did not show a main effect of distance, *F(1*,*16) =* 0.74, *p =.*40, but did show a main effect of length, *F(2*,*32) =* 19.5, *p <.*001, *ηp2 =* 0.55. A planned comparison of the linear size component was conducted to test the compliance of the JND with Weber’s Law. As expected, the test showed that the JND increased linearly with object length, *F(1*,*16) =* 28, *p <.*001, *ηp2 =* 0.63, in line with Weber’s Law.

More importantly, there was no interaction between distance and the linear component of size, *F(1*,*16) =* 1.2, *p =*.27. This indicates that the linear pattern of the JND was similar in both conditions. As before, a Bayesian repeated-measures ANOVA revealed moderate support for the null hypothesis regarding the interaction between distance and size, with the model including the interaction being approximately 8.5 times less likely than the best-fitting model that only included object size (BF₀₁ = 8.534). Individual Bayesian paired-samples t-tests testing the effect of distance at each object size were consistent with this result, providing anecdotal to moderate evidence for the null hypothesis (BF₀₁ = 3.59, 3.46, and 2.14 for the three sizes, respectively).

Also, as in the previous experiments, a specific comparison between standards with (3.5 cm close, 6.5 cm far), and without (3.5 cm far, 6.5 cm close) the same retinal size was conducted. As before, the difference in JND was not significant, *t(16)* = 0.6, *p* =.51, indicating that differences in retinal size were not the (main) factor determining the JND.

Unlike Experiment 1–2, an analysis of the PSE using a repeated-measures ANOVA did not result in a significant effect of viewing distance, *F(1,16) = 1.1*, *p* =.30. There was a main effect of size, *F(2, 32) = 2248.5*, *p* <.001, * ηp2 = 0.99*. However, there was also an unexpected interaction between distance and size, *F(2,32) = 6.4*, *p* <.001, *ηp2 = 0.28*. To further explore this interaction, a within-subject contrast tests for each standard was performed. The tests did not show significant effects of distance for the smallest, *t*(16) = 0.3, *p* =.71 and largest standards, *t*(16) = 0.1, *p* =.90. There was a significant effect of distance for the medium-sized object, however, which was perceived as smaller at the near as compared to the far distance, *t*(16) = 2.1, *p* =.04 (48.7, 50.4 mm, for near and far presentation, respectively).

Finally, to examine whether PSE predicts JNDs size, a multiple linear regression was conducted with PSE, distance, and the interaction term as independent factors. The model, *F*(3, 101) = 11.94, *p* <.001, *R²* = 0.262, adjusted R² = 0.240, showed a significant effect of PSE, *b* = 0.044, t(101) = 3.88, *p* <.001, indicating that higher perceived size was associated with increased JNDs. In contrast, viewing distance was not a significant predictor, *b* = − 0.304, t = − 0.36, *p* =.720, nor was the interaction between PSE and distance, *b* = 0.009, t = 0.55, *p* =.581.

## Discussion

In this series of experiments, classic psychophysical methods were employed to investigate whether distance-related effects on perceptual judgments, previously observed in 2D environments, could extend to 3D environments that included rich binocular and monocular depth cues. The results of our four experiments did not support this hypothesis. Visual discrimination of size, measured by just-noticeable differences (JNDs), was found to be solely influenced by the size of the object, irrespective of noticeable differences in viewing distance, within and beyond the boundaries of peripersonal space.

It is important to note that although we did not find an effect of distance on the JND, changes in viewing distance did affect the perceived size of the object, or PSE. As mentioned earlier, these findings align with previous work, which shows that perceived size tends to vary with distance (Komoda & Ono, 1974; Leibowitz & Moore, 1966; Leibowitz et al., 1972; Ono et al., 1974; Oyama, 1974). Note, however, that the effect of distance on PSE was not extended to experiment 3, which employed a different psychophysical method, as well as when the standards were 3D objects and the participant’s head was not fixed (Experiment 4). Nevertheless, the results of Experiments 1–2 suggest that while the JND is unaffected by viewing distance, overall biases in the perceived size of objects can occur, particularly when head movements are minimized. This indicates that while our ability to detect change in magnitude depends on a high-level visual representation of objects, it is at least partially independent of conscious size perception (PSE).

In this context, it is important to note that while PSE and JNDs are related measures, they are believed to reflect dissociable functions (Baird & Noma, 1978; Marks & Algom, 1998). Supporting this interpretation, previous studies have shown complementary patterns of changes in JNDs without corresponding changes in PSE (for some recent examples, see Hadad & Schwartz, 2019; Namder et al., 2016; Paire et al., 2023). For example, Namder et al. (2016) found that JNDs for the same stimuli varied depending on the range of other standards tested for resolution, suggesting that perceptual resolution can be influenced by context independently of perceived size. The exact nature of the interaction between JND and PSE should be examined further in future research.

The results of the current study differ from those reported in recent work that used illusory 2D depth cues, where better discrimination was observed for objects perceived as closer to the observer (Ahsan et al., 2022; Blini et al., 2018; also see, Ahsan et al., 2025; Britt et al., 2025; Dureux et al., 2021; Haponenko et al., 2024; Zafarana et al., 2024). In contrast to these studies, the perceptual resolution in the current work was consistent across both (3d) viewing conditions. A potential explanation for this discrepancy is that the presentation of stimuli within a size-contrast illusion configuration has also affected their perceived size, leading to differences in JNDs and perceptual judgments.

However, perhaps a more intriguing possibility relates to the dimensionality of the stimuli themselves. Obtaining size consistency may be significantly easier for stimuli viewed in a 3D environment, which includes richer binocular and monocular cues (as well as information about shape and size in the case of 3D objects). Indeed, a growing body of evidence suggests that 2D and 3D stimuli are processed differently (Freud & Ganel, 2015; Freud et al., 2018; Holmes & Heath, 2013; Korisky & Mudrik, 2021; Ozana & Ganel, 2019, 2020a, 2020b; Snow et al, 2011; Snow & Culham, 2021; Yildiz et al., 2024). Notably, size constancy has recently been shown to improve in virtual environments that include pictorial depth cues compared to those without, and to be more effective for linear perspective cues than for texture-based depth cues alone (Yildiz et al., 2024). The “dimensionality” of stimuli used in vision research may play a far more significant role in shaping perceptual response than previously thought.

Considering the broader literature on near-space advantage, the current results may support the idea that this phenomenon, well-documented in attentional tasks such as target detection and localization (Britt et al., 2025), reflects higher-level cognitive mechanisms involving response bias rather than low-level perceptual processes that influence how objects are represented and perceived (Bufacchi & Iannetti, 2018). Future research should explore this possibility further across additional object dimensions, such as shape and color. The use of complementary methods beyond the block design of standard psychophysical measurements (Marks & Algom, 1998; Marks & Gescheider, 2002) should also be considered in future work, while also accounting for noise-related issues associated with randomized presentation (Rolland et al., 2002).

In previous studies on the perceptual judgment of size, participants were typically asked to estimate the size of objects under fixed viewing conditions, where the viewing distance and retinal image size remained constant throughout the experiment. This correlation raised another intriguing question that the current study was able to address: Does the size of JND depend on retinal size or perceived size? By using dedicated comparisons that dissociated perceived size from retinal size, it was revealed that perceived size reliably determined the JND. Thus, the JND remained invariant whether the same pair of stimuli subtended the same retinal-image size (the near 3.5 cm and far 6.5 cm objects) or different retinal-image sizes on the retina (near 6.5 cm and far 3.5 cm).

At first blush, these findings do not appear to be consistent with previous correlational research that linked neural events in early sensory areas of the monkey to human performance in comparative judgment tasks as well. An extensive literature has shown that the neural response pattern of early sensory neurons can predict psychophysical behavior in a range of perceptual judgment tasks (for reviews, see Johnson et al., 2002; Jung, 1972; Parker & Newsome, 1998). On the basis of these observations, it would have seemed reasonable to assume that our ability to discriminate size also depends on early sensory coding, such as the differential activation of neurons related to the image projected on the retina. The current results, however, show that it is the perceived size of the object that modulates the observer’s visual resolution.

In this context, it is interesting to note that several fMRI studies have demonstrated that the extent of activation in human V1 reflects the perceived and not retinal size of the visual stimulus (Murray et al., 2006; Sperandio et al., 2012). Although this result might seem surprising, it is important to remember that the vast majority of studies in animals that have investigated the retinotopic organization of V1 have used a fixed viewing distance (typically 57 cm, whereby 1 cm on the screen is 1° of visual angle). Moreover, the time constant of the BOLD signal in fMRI is so slow that it cannot detect any changes in V1 activity that might occur after bottom-up input reaches V1. A recent EEG study, however, showed that, although the initial signal in early visual areas reflects the retinal image size of a visual stimulus, 150 ms later the signal reflects its perceived size (Chen et al., 2019a, b). The timing in the change from retinal image size to perceived size in the EEG signal presumably reflects the arrival of recurrent information from higher-order visual areas that compute viewing distance from a wide range of depth cues. The fact that activity in the cerebral visual pathways, even the early retinotopic areas such as V1, comes to reflect the perceived size of an object may explain why the resolution of the judgments of object length in our experiment is tied to the perceived, not the retinal image size of the objects.

It should also be noted that the viewing distances used in the current design (35 and 65 cm) corresponds with previous literature on peripersonal space (see review by Serino, 2019), as well as to ensure a meaningful change in the visual angle of same-sized objects projected onto the retina (e.g., 5.72° and 10.6° for the 6.5 cm object at the near and far viewing distances, respectively). Yet, it is possible to use the current design to test for perceived size and JND at larger distances from the observer. At larger distances from the observer, the unavailability of reliable distance cues could interfere with size constancy. Moreover, there might not only be greater trial-to-trial variability in perceived size but also larger individual differences than what was tested here. Future work could investigate whether poorer size constancy also entails poorer resolution of size and/or the extent to which individual differences in perceived size correlate with perceptual resolution.

## Supplementary Information

Below is the link to the electronic supplementary material.

Supplementary File 1 (12.0KB)

## Data Availability

The data is provided in a supplementary information file.
